# Small-Molecule Bi-DOTA Complex for High-Performance CT and Spectral CT Bioimaging

**DOI:** 10.3389/fonc.2022.813955

**Published:** 2022-02-18

**Authors:** Guidong Dai, Yu Zhang, Ximei Wang, Xingyu Wang, Juan Jia, Fei Jia, Lu Yang, Chunmei Yang

**Affiliations:** ^1^ Department of Radiology, The Affiliated Hospital of Southwest Medical University, Nuclear Medicine and Molecular Imaging Key Laboratory of Sichuan Province, Luzhou, China; ^2^ Department of Medical Imaging, Southwest Medical University, Luzhou, China

**Keywords:** Bi-DOTA complex, CT imaging, spectral CT imaging, small molecular, CT contrast agent

## Abstract

**Objectives:**

It is necessary to develop a high-performance and biocompatible contrast agent to accurately diagnose various diseases *via in vivo* computed tomography (CT) imaging. Here, we synthesized a small molecular Bi-DOTA complex as a high-performance contrast agent for *in vitro* and *in vivo* CT bioimaging.

**Materials and Methods:**

In our study, Bi-DOTA was fabricated through a facile and one-pot synthesis strategy. The formed Bi-DOTA complex was characterized *via* different techniques. Furthermore, Bi-DOTA was used for *in vitro* and *in vivo* CT bioimaging to verify its X-ray attenuation ability, especially *in vivo* kidney imaging, gastrointestinal tract CT imaging, and spectral CT imaging.

**Results:**

A small molecular Bi-DOTA complex with a molecular mass of 0.61 kDa was synthesized successfully, which exhibited outstanding dispersion, good biocompatibility, and superior X-ray attenuation ability. Meanwhile, we showed that the obtained contrast agent was quite biocompatible and safe in the given concentration range as confirmed by *in vitro* and *in vivo* cytotoxicity assay. Also, the proposed contrast agent can be rapidly excreted from the body *via* the urinary system, avoiding the potential side effects caused by long-term retention *in vivo*. Importantly, Bi-DOTA was successfully used in high-quality *in vitro* CT imaging, *in vivo* kidney imaging, gastrointestinal tract CT imaging, and spectral CT imaging.

**Conclusions:**

These superiorities allowed Bi-DOTA to be used as an efficient CT contrast agent and laid down a new way of designing high-performance CT contrast agents with great clinical transformation potential.

## Introduction

Computed tomography (CT) is one of the most widely used imaging techniques in diagnostic medicine due to its high resolution, unlimited tissue penetration, and cost effectiveness (1–5). The emerging spectral CT is the newest technique in a series of CT imaging advances, which has been used in many studies and clinical imaging. However, the use of a large amount of contrast agents is often required for accurate diagnosis of various diseases because noncontrast CT has lower sensitivity and soft tissue contrast (6).

Currently, contrast agents can be divided into two major categories: small molecular agents (iodinated molecules and lanthanide-chelates) and nanostructures containing high-Z elements (7), in which iodinated compounds are widely used as CT contrast agents. Nevertheless, clinically used iodinated small molecular agents (e.g., iohexol, ioversol, and iopamidol) suffer from low sensitivity, poor spectral CT, potential allergy, and renal toxicity (6, 8, 9). In addition, they are rapidly cleared *via* kidney, resulting in short circulation lifetime that limits their applications for CT angiography and CT perfusion imaging. Recently, it has been reported that the iodinated contrast agent is incorporated into polymers or polymer nanoparticles to extend the blood circulation time of the contrast agent, thereby realizing effective blood pool imaging (10–13). However, it is another obvious disadvantage that iodine has relatively low K-edge of (33 keV), which has a higher potential for damaging tissues (14). Furthermore, many studies indicated that lanthanide-chelates (such as Gd-DTPA and Nd-DTPA) not only enable enhanced magnetic resonance imaging (MRI) but also can serve as CT contrast agents (15–22). Nevertheless, considering the larger dosage, the toxicity, and the high cost of lanthanide element, their applications are seriously limited in CT imaging. In the past decades, nanoparticulate CT contrast agents that comprise high-Z elements [e.g., gold (23, 24), argentine (25, 26), bismuth (27, 28), hafnia (29, 30), ytterbium (31, 32), tantalum (33, 34), holmium (35), and rhenium (36)] have been proven to own a great potential for CT imaging. Despite good imaging effects and acceptable safety profiles, these nanoparticulate CT contrast agents still have some disadvantages, including intrinsic long-term retention, difficulty in biodegradation *in vivo*, slow excretion from the body and the accompanying side effects, the high cost, and complex synthesis techniques (16, 17, 37, 38) compared with conventional iodine-based contrast agents. Therefore, it is critical but challenging to find a high-performance, biocompatible, low-cost, and safe contrast agent for enhanced CT and spectral CT imaging, as it may assist accurate diagnosis of various diseases and guide clinical treatment strategies.

Bismuth (Bi), the 83rd element in the periodic table, is considered a biosafety element and one of the least expensive heavy metal elements, which shows a much more sensitive CT imaging capability because of the higher X-ray attenuation coefficient than iodine (16, 17, 38, 39) (5.74 vs. 1.94 cm^2^ g^−1^, 100 keV). Therefore, Bi is a promising element for developing high-performance CT contrast agents. Currently, it is reported that some Bi-based materials, e.g., Bi (27, 40), Bi_2_S_3_ (41, 42), Bi_2_Se_3_ (37, 43), Bi-diethylene triamine pentaacetate acid (7), and Gd-PEG-Bi NPs (40) have been constructed as new CT contrast agents (43, 44). However, there is still a paucity of Bi-based contrast agents receiving significant attention for clinical application. Therefore, there is currently a great need for developing Bi-based CT contrast agents with great clinical translation potential.

At present, chelate ligands, such as 1,4,7,10-tetraazacyclododecane-1,4,7,10-tetraacetic acid (DOTA) and its derivatives, have been explored to fabricate metal-ligand complexes for biological applications, e.g., Gd-DOTA (45), Gd-5-HT-DOTAGA (46), and Gd-DO3A (1,4,7,10-tetraazacyclododecane-1,4,7,-triacetic acid) (47). Here, the aim of our study was to combine Bi with the derivative of DOTA for fabricating a high-performance CT and spectral CT bioimaging contrast agent.

## Experimental Section

### Chemicals and Materials

All chemical reagents were obtained from the commercial supply and used without further purification. Bi(NO_3_)_3_·5H_2_O, NaOH, and 1,4,7,10-tetraazacyclododecane-1,4,7,10-tetraacetic acid (DOTA) were purchased from Shanghai Macklin Biochemical Co., Ltd. (Shanghai, China). Roswell Park Memorial Institute-1640 (RPMI-1640) medium, Dulbecco’s minimum essential medium (DMEM), high sugar medium, and fetal bovine serum (FBS) were purchased from Sigma-Aldrich (St. Louis, MO, USA). Penicillin-streptomycin solution, trypsin digestion fluid, and PBS buffer (pH 7.2–7.4) were purchased from Beyotime (Haimen, China). The Fourier transform infrared (FT-IR) spectra of Bi-DOTA were carried out on an IRAffinity-1S spectrometer (Shimadzu, Japan). The Bi content in the isolated organ was measured using inductively coupled plasma optical emission spectrometry (ICP-OES, Agilent, Santa Clara, CA, USA). Test conditions were as follows: emission power 1.0 kW, plasma gas flow 15 L/min, auxiliary gas flow 1.5 L/min, argon carrier, detection mode for axial observation, and linear calibration. Matrix-assisted laser desorption ionization (MALDI) time of flight (TOF)-mass spectrometer (MALDI-TOF-MS) of Bi-DOTA (without the treatment of NaOH) was obtained on ultraflextreme MALDI-TOF (Bruker, Ettlingen, Germany). ^1^H NMR spectra and ^13^C NMR spectra were measured on a Bruker Biospin AV400 (400 MHz) instrument. Chemical shifts are reported in parts per million (ppm) and are referenced to tetramethylsilane.

### Synthesis of Bi-DOTA Complex

**Scheme 1:**
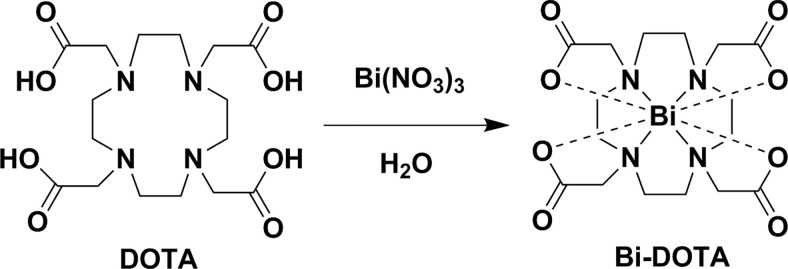
Schematic representation of the synthesis of Bi-DOTA.

For synthesis of the Bi-DOTA complex, Bi(NO_3_)_3_·5H_2_O (395 mg, 1 mmol) was slowly added to a solution of DOTA (404 mg, 1 mmol) in deionized water (20 ml) and stirred until forming a transparent solution (**Scheme 1**). The solution was then stirred for 24 h at 85°C; the obtained Bi-DOTA solution was treated with 0.4 M NaOH to adjust the pH value to 7.2, and the product of Bi-DOTA was freeze dried to give a bright purple solid Bi-DOTA. The NMR spectra of Bi-DOTA are given as follows: ^1^H NMR (400 MHz, D_2_O) *δ* 4.17 (s, 4H), 3.85 (s, 4H), 3.34 (s, 12H), 3.17 (s, 4H). ^13^C NMR (101 MHz, D_2_O) *δ* 178.35, 58.41, 54.48, 49.91. MALDI-TOF-MS Calcd for: C_16_H_25_BiN_4_O_8_^+^ ([M+H]^+^): 610.15. Found: 615.10. IR (KBr, ν): 3,456, 3,398, 2,978, 2,877, 1,658, 1,384, 1,083, 925, 833, and 709 cm^−1^.

### The Colloidal Stability of Bi-DOTA

To evaluate the colloidal stability of Bi-DOTA, Bi-DOTA was dissolved in various media including PBS, FBS, and DMEM for 14 days at 37°C.

### Cell Culture and Cytotoxicity Assessment

LO2 cells, MCF-10A cells, human intrahepatic biliary epithelial cells (HIBEC), and human umbilical vein endothelial cells (HUVEC) were obtained from ATCC (Gaithersburg, MD, USA). These cells were cultured in DMEM solution respectively in a cell incubator at 37°C and 5% CO_2_ condition. The cell cytotoxicity *in vitro* was measured by 3-(4,5-dimethylthiazol-2-yl)-2,5-diphenyltetrazolium bromide (MTT) assay. Four cells were seeded into a 96-well cell culture plate at 5,000/well and then incubated for 24 h at 37°C and 5% CO_2_, respectively. DMEM solutions of Bi-DOTA with different sizes (concentrations: 0, 25, 50, 100, 200, 400, and 600 mg/L) were added to the wells. All cells were then incubated for 24 h at 37°C and 5% CO_2_, and the cell viability was calculated using a typical MTT assay.

### Toxicity Assessment *In Vivo*

To investigate the *in vivo* toxicity of Bi-DOTA, the mice (*n* = 12) were sacrificed at different time points postinjection (7 and 14 days, respectively). For comparison, the mice (*n* = 3) injected with glucose solution were used as controls and sacrificed at 14 days. The major organs (heart, spleen, liver, lung, kidney stomach, intestine, and colon) were carefully harvested, weighed, and fixed with 4% formalin solution. Finally, pathological section and hematoxylin and eosin (H&E) staining analysis were carried out to assess the *in vivo* toxicity of Bi-DOTA. In addition, the body weight changes of the mice have been measured every 2 days.

Some biochemical indicators of these mice including the liver function indicators are total protein (TP), albumin (ALB), aspartate alanine aminotransferase (ALT), and total bile acid (TBA), and typical biomarkers of kidney function are serum creatinine (CREA) and urea nitrogen (BUN) at different time points (7 and 14 days, *n* = 3 for each group) after the injection of different concentrations of Bi-DOTA (0.1 and 0.2 mol/L) were measured.

### Spectral CT Imaging *In Vitro*

To compare the X-ray absorbance ability and spectral CT performance of Bi-DOTA with iohexol, 2 ml of various concentrations (0, 0.0125, 0.025, 0.05, and 0.1 mol/L) of Bi-DOTA and iohexol (Yangzijiang Pharmaceutical Group, Nanjing, China) were added into a tube to achieve the *in vitro* CT phantom imaging, which were performed on the spectral CT (IQON Spectral CT, Philips, Amsterdam, Netherlands). The parameters were set as follows: field of view 150 × 150 mm, slice thickness 0.4 mm, tube current 100 mA, and tube voltage 120 kV. Furthermore, monochromatic images and spectral CT value curves were acquired at the photon energy range of 40–200 keV with a 5-keV increment using Bi-DOTA and iohexol.

### Spectral CT Imaging *In Vivo*

The 6-week-old BABL/C male mice (average body weight: 17 g) were purchased from Dashuo Animal Technology Limited, Jianyang, China. All mice were housed at 20°C–25°C and 50% ± 5% humidity with a 12-h light-dark cycle and an access to food and water. All procedures and animal experiments were approved by the Animal Ethnical Committee of Southwest Medical University and conducted in accordance with state regulations. Furthermore, all CT scanning were performed on spectral CT (IQON Spectral CT, Philips, Netherlands) in our study.

After a week, the mice were anaesthetized using a small animal ventilator with isoflurane (1.5%–2.5%, 0.8 ml/min oxygen flow rate) and then scanned before and after the injection of 200 μl Bi-DOTA (0.1 and 0.2 mol/L, respectively) *via* the tail vein at different time points (0, 1 min, 5 min, 30 min, 1 h, 1.5 h, 3 h, 6 h, and 12 h). The parameters were set as follows: field of view 150 × 150 mm, slice thickness 0.4 mm, tube current 100 mA, and tube voltage 120 kV. Subsequently, virtual monochromatic images of these mice were obtained at the photon energy range of 40–200 keV with a 10-keV increment. The 3D reconstruction was carried out on a Philips Intellispace Portal Workstation. For comparison, the intravenous spectral imaging of mice was also carried out using iohexol at an equivalent dose and monochromatic energy.

Moreover, to further confirm the excellent CT imaging ability of Bi-DOTA, the gastrointestinal tract (GI tract) CT imaging of mice was performed after the oral administration of Bi-DOTA (200 μl, 0.1 mol/L, and 0.2 mol/L, respectively) at different time points (0, 1 min, 5 min, 30 min, 1 h, 1.5 h, 3 h, 6 h, and 12 h). The scanning parameters were set as follows: field of view 150 × 150 mm, slice thickness 0.4 mm, tube current 100 mA, and tube voltage 120 kV. Similarly, virtual monochromatic images of these mice were also acquired at the photon energy range of 40–200 keV with a 10-keV increment, and the 3D reconstruction was also performed. As control, the GI tract imaging of mice was further carried out using iohexol at an equivalent dose and monochromatic energy.

## Results

### Synthesis and Characterization of Bi-DOTA

The Bi-DOTA was synthesized through a chelation of the Bi^3+^ and DOTA, as presented in **Scheme 1**. The DOTA and Bi-DOTA were analyzed by FTIR. As demonstrated in **Figure S1**, for pure DOTA, the peak at 1,678 cm^-1^ is ascribed to the stretching vibration of C=O (–COOH). While in Bi-DOTA, the stretching and deformation vibration band of 1,678 cm^−1^ disappeared and a new vibration peak at 1,600 cm^−1^ was observed, validating the coordination of COO^−^ with Bi^3+^. To further reveal the formation of Bi-DOTA, we have performed ^1^H NMR and ^13^C NMR spectroscopic analyses. As shown in **Figures S2** and **S3**, ^1^H NMR and ^13^C NMR result further validates the formation of Bi-DOTA. In addition, we have compared the ^1^H NMR (400 MHz, D_2_O) spectra of DOTA and Bi-DOTA in **Figure S4**. These results show significant changes when the –COOH of DOTA is coordinated with Bi(III) compared with DOTA. The peaks around 4.17, 3.85, 3.34, and 3.17 ppm for Bi-DOTA can be attributed to the coordination of the lanthanide center, while the peaks for DOTA are at 3.63 and 3.16 ppm (**Figure S4**). The content of Bi element in Bi-DOTA was quantified by ICP-OES to be 31.66%, and the peak at m/z 610.10 in MALDI-TOF-MS of the synthesized chelate (without NaOH) was assigned to Bi-DOTA structure ([M+H]: calculated for 610.15), further demonstrating the formation of Bi-DOTA chelate (**Figure S5**). Furthermore, stability test showed that Bi-DOTA possessed excellent solubility and stability in different media, such as PBS, FBS, and DMEM (**Figure S6**). Their excellent colloidal stability was further demonstrated by the absence of precipitates or aggregates in their aqueous solutions during 14 days of storage.

### Cell Culture and Cytotoxicity Assessment

After incubation of LO2 cells, MCF-10A cells, HIBEC, and HUVEC with Bi-DOTA at concentrations of 0, 25, 50, 100, 200, 400, and 600 mg/L for 24 h, MTT assay was used to evaluate the cell viability. The results in **Figure S7** demonstrated that Bi-DOTA had good biocompatibility, and no serious cytotoxicity toward the above four cells was observed in the range of 0–600 mg/L, in which the cell viability was higher than 80% and even treated with 600 mg/L of Bi-DOTA. These results demonstrated the low cytotoxicity of Bi-DOTA considering its rapid renal clearance as a small molecular contrast agent.

### Toxicity Assessment *In Vivo*

**Figure 1:**
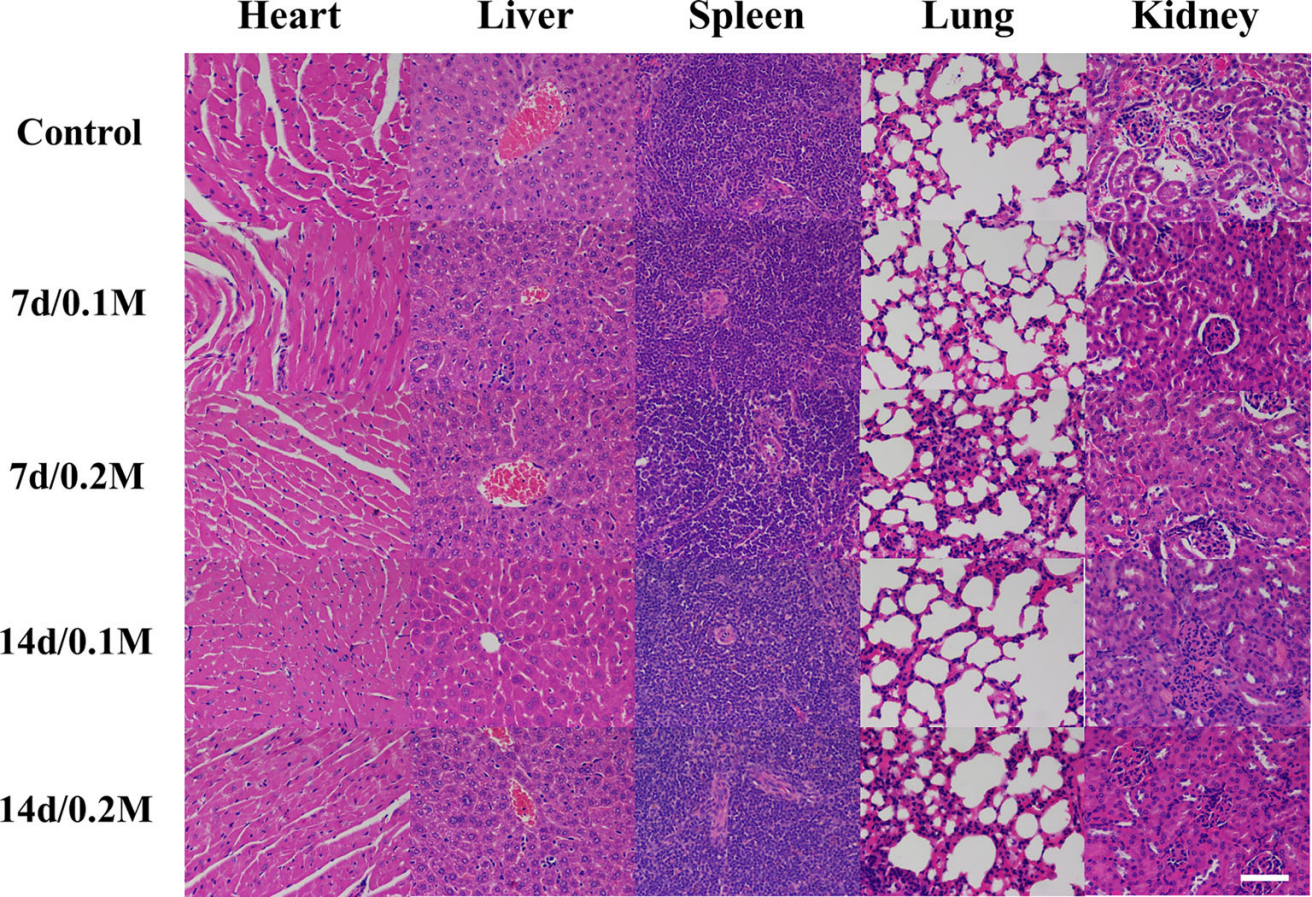
HE staining of main organs (heart, liver, spleen, lung, and kidney) after intravenous administration of 200 μl of different concentrations of Bi-DOTA (0.1 and 0.2 M) after different time points (7 and 14 days) (scale bar: 200 μm).

**Figure 2:**
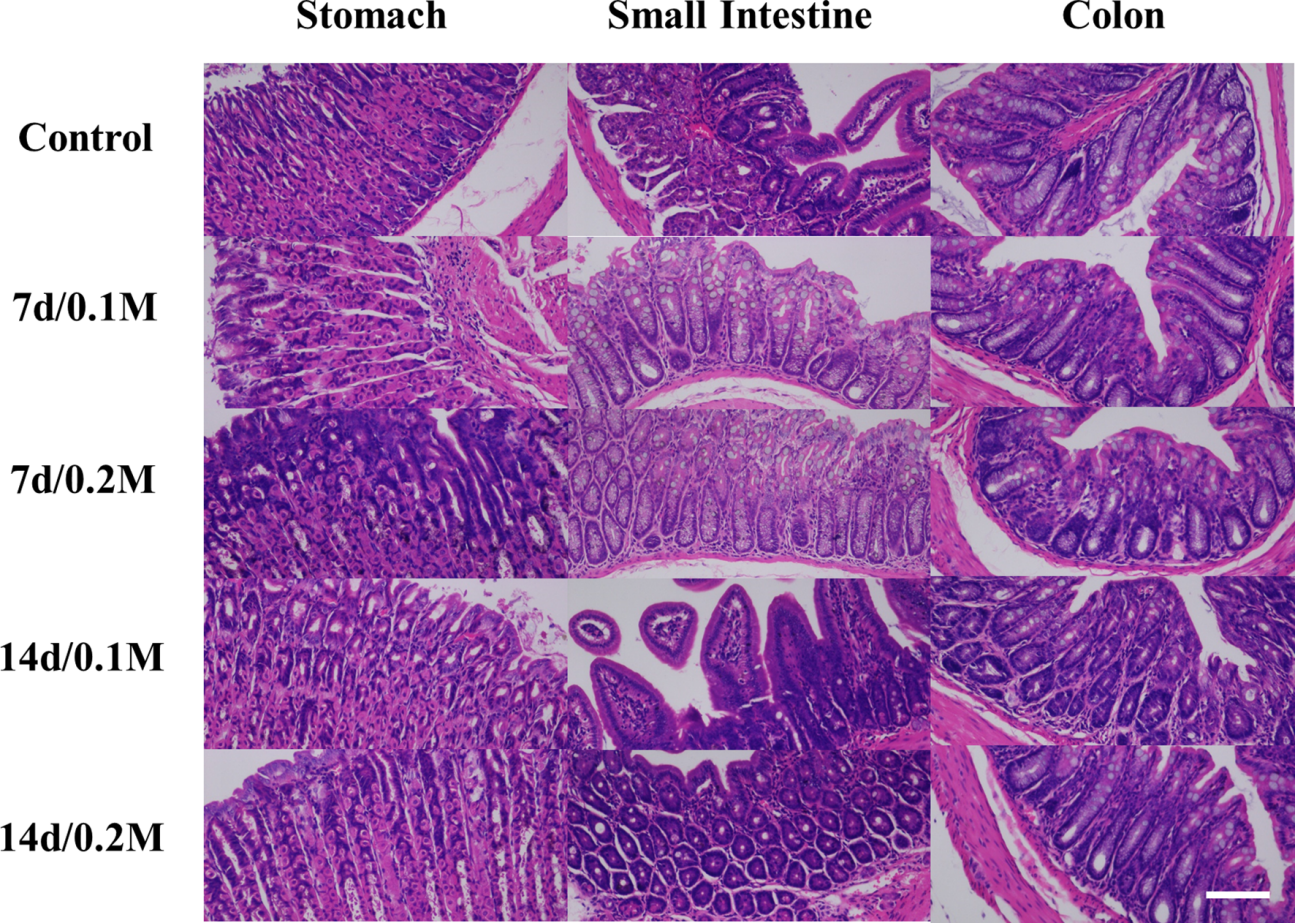
HE staining of GI tract (stomach, small intestine, and colon) after oral administration of 200 μl of different concentrations (0.1 and 0.2 M) of Bi-DOTA after different time points (7 and 14 days) (scale bar: 200 μm).

The histopathological analysis was employed to evaluate the *in vivo* toxicity of Bi-DOTA. No death occurred and no abnormal activity was found in the mice within 7 days/14 days after they were injected with Bi-DOTA *via* the tail vein. For these mice, H&E staining indicated the microstructures of various organs including myocardial striation and muscle fibers, hepatic lobules and the hepatocytes, lymphoid follicles and germinal center, alveoli, and collecting ducts were all exhibiting normal morphology and arranged regularly at different time points (**Figure 1**). For the mice treated with Bi-DOTA orally, H&E analysis indicated that the mucosal, submucosal, and muscular structures of GI tract were all clear and did not show obvious histopathological damages after the administration of different concentrations of Bi-DOTA at different time points (**Figure 2**). In conclusion, no tissue necrosis and inflammatory response were observed compared with the control group. Furthermore, the changes in body weight in different treatment groups were measured every 2 days (**Figure S8**). These results indicated that the small molecule Bi-DOTA complex did not significantly reduce body weight, indicating its lower toxicity to normal body tissues.

In addition, clinic biochemical indicators were also measured before and after intravenous injection of different concentrations of Bi-DOTA at different time points. The vital indicators of liver function (TP, ALB, ALT, and TBA) and typical biomarkers of kidney function including CREA and BUN at 7 and 14 days all demonstrated no significant differences compared with the control group (**Figure S9**).

### Spectral CT Imaging *In Vitro*

**Figure 3:**
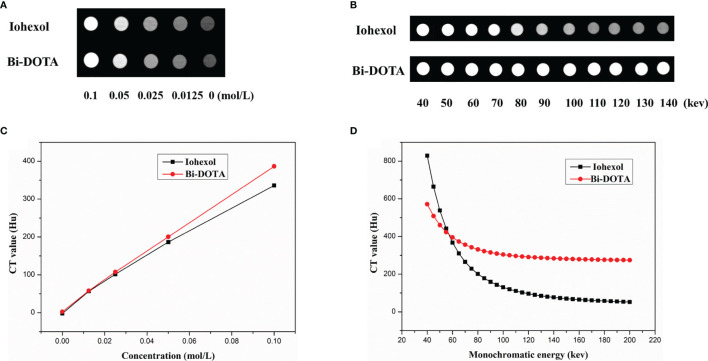
**(A)** CT phantom imaging under different concentrations at 120 kV. **(B)** A linear relationship between CT values and the concentrations of both Bi-DOTA and iohexol. **(C)** Spectral CT monochromatic images under different energies using Bi-DOTA and iohexol. **(D)** Spectral CT value curves of Bi-DOTA and iohexol at different energies.

First, the X-ray attenuation efficiency of Bi-DOTA was assessed compared with iohexol. As shown in **Figures 3A, B**, the increase in CT values showed a linear relationship with the concentrations of both Bi-DOTA and iohexol. Also, the CT values of Bi-DOTA were higher than iohexol at equivalent concentrations at the tube voltage of 120 kV, and the X-ray attenuation coefficients of Bi-DOTA were more eminent than iohexol.

To investigate spectral CT performance of Bi-DOTA, monochromatic images (**Figure 3C**) and spectral CT value curves (**Figure 3D**) were acquired using Bi-DOTA and iohexol. We can see that the CT values of Bi-DOTA and iohexol decreased with the increase of X-ray energy (40–200 kev). Under lower energy level (such as 50–60 kev), it was difficult to make a discrimination between Bi-DOTA and iohexol. Nevertheless, the CT values of iohexol declined sharply with the increase of energy (70–200 kev), while the CT values of Bi-DOTA only showed a slight decrease due to its outstanding X-ray attenuation capability (**Figure 3D**). Such appealing X-ray attenuation characteristic endues Bi-DOTA with excellent contrast effect for disease diagnosis compared with iohexol, especially in a higher monochromatic energy.

### Spectral CT Kidney Imaging *In Vivo*

**Figure 4:**
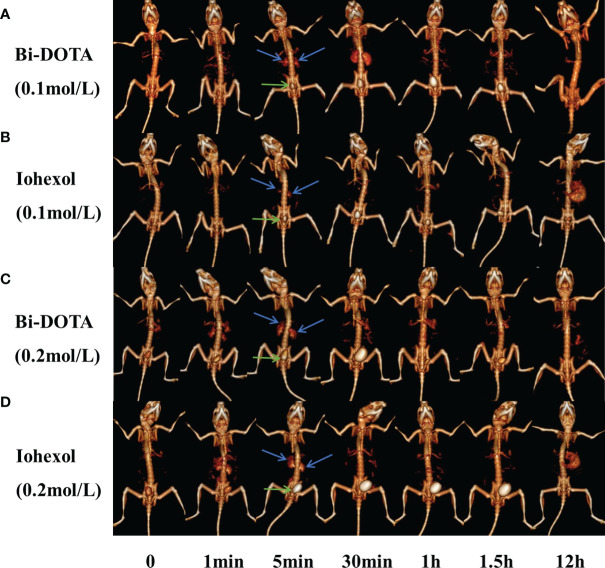
*In vivo* CT urography imaging using Bi-DOTA and iohexol (blue arrows represent kidney and green arrows represent bladder). CT imaging after intravenous administration of **(A)** 0.1 mol/L Bi-DOTA, **(B)** 0.1 mol/L iohexol, **(C)** 0.2 mol/L Bi-DOTA, and **(D)** 0.2 mol/L iohexol.

First, 200 μl of 0.2 mol/L Bi-DOTA were intravenously injected into the BABL/C male mice and the same dosage of iohexol as a control. In the Bi-DOTA group, an opacified renal collecting system could be observed when reaching 1 min and became clear gradually until 5 min. The enhanced CT signal then decreased after 5 min and gradually returned to nonenhancement 30 min later (**Figure 4C**). Meanwhile, the CT value of the bladder is gradually augmented, indicating that Bi-DOTA was metabolized *via* the urinary system and can be rapidly eliminated from the body. For the mice which experienced an intravenous injection of 200 μl of iohexol with equivalent concentration, their collecting system of kidney and bladder was also lighted up at the similar time points (**Figure 4D**). However, CT values in region of interest (kidney) after administration of Bi-DOTA at 1, 2, 3, 4, and 5 min were higher than those after the treatment with iohexol (**Figure S10** **B**). What this suggested was that the CT enhancement effect of Bi-DOTA exceeded that of iohexol at the same concentration.

In order to prove the excellent X-ray attenuation ability, we further performed CT imaging using Bi-DOTA at a lower concentration (0.1 mol/L) (**Figure 4A**). We can see that a renal collecting system appeared when reaching 5 min and became clear gradually until 30 min. The enhanced CT signal then decreased after 30 min and gradually returned to nonenhancement 1 h later. In contrast, a renal collecting system displayed poorly and showed a much lower contrast enhancement in the control group due to the weaker X-ray attenuation ability of iohexol (**Figure 4B**). Although the performance of the kidney imaging at the lower concentration (0.1 mol/L) was inferior to that at the higher concentration (0.2 mol/L), Bi-DOTA at the lower concentration had still more obvious contrast enhancement compared with iohexol in our study (**Figure S10A**), which demonstrated that Bi-DOTA had a great potential to be employed as a high-performance CT control agent.

**Figure 5:**
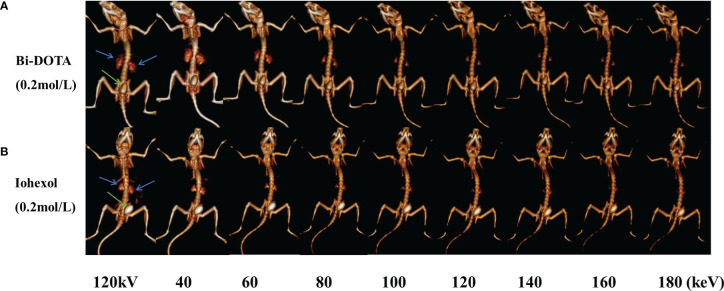
*In vivo* spectral CT urography imaging under different energies using 0.2 mol/L Bi-DOTA **(A)** and iohexol **(B)** (blue arrows represent kidney and green arrows represent bladder).

Furthermore, virtual monochromatic images of mice were also obtained at the photon energy range of 40–200 keV with a 10-keV increment. **Figure 5** shows the CT signal of a renal collecting system by Bi-DOTA (**Figure 5A**) and iohexol (**Figure 5B**) in monochromatic images decreased gradually with the rise of energies. It can be seen that both signals of Bi-DOTA and iohexol in the kidney at around 40 keV were the highest. In addition, the enhanced signals of Bi-DOTA-delineated kidney were higher than that of iohexol-delineated kidney at the same energy, which indicated that Bi-DOTA may have a more outstanding performance of spectral CT imaging compared with iohexol. Furthermore, compared with iohexol, Bi-DOTA had lower molecular weight (611.16 kDa vs. 821.13 kDa), which is expected to have the higher renal clearance rate.

### Spectral CT GI Tract Imaging *In Vivo*

**Figure 6:**
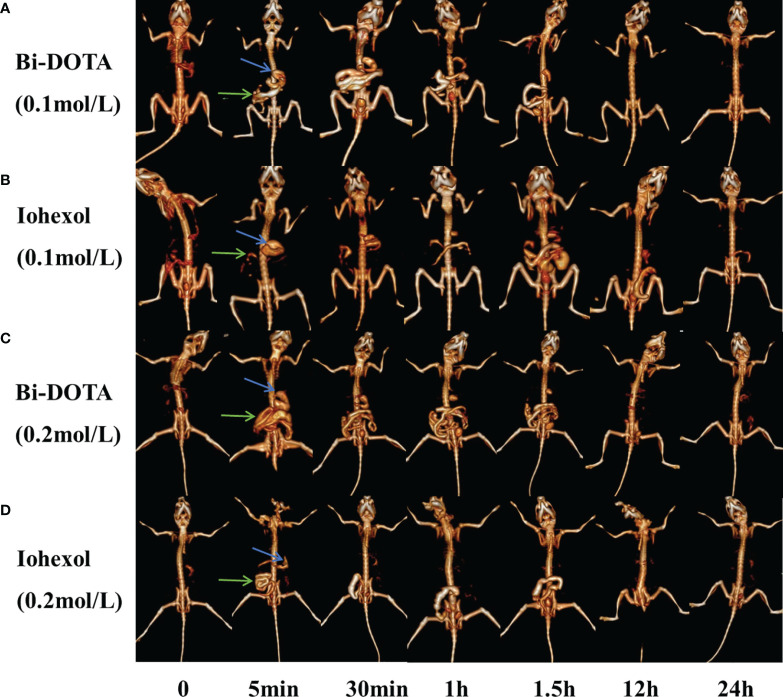
*In vivo* GI tract CT imaging after oral administration of **(A)** 0.1 mol/L Bi-DOTA, **(B)** 0.1 mol/L iohexol, **(C)** 0.2 mol/L Bi-DOTA, and **(D)** 0.2 mol/L iohexol (blue arrows represent stomach and green arrows represent small intestine).

To further confirm the excellent spectral CT imaging ability, the CT imaging of the upper and lower GI tract was carried out using Bi-DOTA (0.2 mol/L) (**Figure 6C**). At 5 min after oral administration of 200 μl of 0.2 mol/L Bi-DOTA, the stomach, duodenum, and proximal jejunum were obviously lighted up due to the filling of the control agent. The signal of the stomach gradually became weaker and more loops of small intestine were delineated 5 min later. At 1 h after oral administration, the whole jejunum and ileum were bright and the signal of the stomach was further weakened. Bi-DOTA clearly outlined the morphology and sequence of the stomach, duodenum, jejunum, and ileum, which would provide accurate diagnosis of the upper GI tract disease or malformation. After 12 h, Bi-DOTA was emptied from the upper GI tract, and then excreted from the body after 24 h.

Indeed, upper and lower GI tract imaging was also performed using a lower concentration of Bi-DOTA (0.1 mol/L) (**Figure 6A**). In a result, we found that the morphology and arrangement of the stomach, small intestine, and large intestine were clearly delineated as similar as the CT imaging efficacy of the high concentration of Bi-DOTA (0.2 mol/L). Namely, it is sufficient to use the concentration of Bi-DTPA at 0.1 mol/L to visualize the GI tract. As a control, upper and lower GI tract imaging of iohexol at a corresponding concentration was also performed (**Figures 6B, D**). However, the perfusion effect of iohexol to image upper GI was inferior to that of Bi-DOTA due to its lower X-ray absorbance ability.

**Figure 7:**
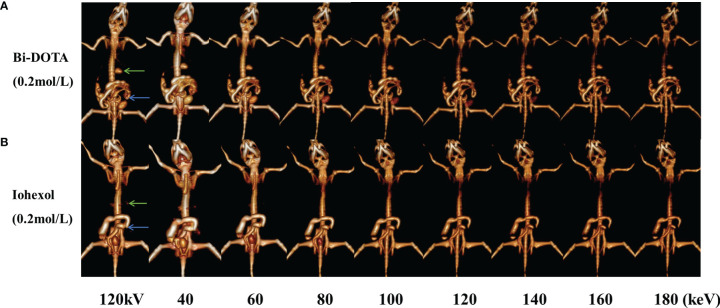
*In vivo* spectral GI tract CT imaging under different energies using 0.2 mol/L Bi-DOTA **(A)** and iohexol **(B)** (green arrows represent stomach and blue arrows represent small intestine).

Meanwhile, the monochromatic images of GI tract at different energies (40, 60, 80, 100, 120, 140, 160, and 180 keV) and CT images under conventional tube voltage of 120 kV were further acquired and reconstructed. The CT signal of GI tract by Bi-DOTA (**Figure 7A**) and iohexol (**Figure 7B**) in monochromatic images decreased gradually with the rise of energies. We can see that both signals of Bi-DOTA and iohexol in the GI tract at around 40 keV were the highest. In addition, the enhanced signals of Bi-DOTA-delineated GI were higher than adjacent tissues and bone at any energies of 40–180 keV. Nevertheless, the CT values of bone and other tissues obviously decreased with the increase of energy, making Bi-DOTA an excellent CT contrast agent.

In conclusion, Bi-DOTA was successfully applied in the upper and lower GI tract in our study, which indicated that it may serve as an excellent CT contrast agent for various biomedical applications compared with the clinical iodinate agent.

## Discussion

To our knowledge, this study has produced a new CT control agent (Bi-DOTA), which owns a simple synthesis process, a low cost, a good biosafety, and a high X-ray attenuation ability across the low and high operating voltage settings. We have made a strong effort to investigate its CT imaging efficacy, finding that it could serve as an excellent CT contrast agent for biomedical applications. What this suggests is that Bi-DOTA is a promising control agent in terms of CT imaging. Accordingly, it may also assist accurate diagnosis of disease and guide the clinic.

Bi has the biggest atomic number among “nonradioactive elements,” thus Bi-based materials hold great attraction as novel contrast agents because of their ultrahigh X-ray attenuation coefficient (15, 17). Also, good biocompatibility, low cost, and “Bi therapy” further make Bi-based materials a favorable candidate for developing CT imaging agents with high performance (16, 48). Currently, it is reported that various investigators have produced a variety of control agents based on Bi for CT imaging, e.g., Bi (27, 40), Bi_2_S_3_ (41), Bi_2_Se_3_ (37, 43), bi-diethylene triamine pentaacetate acid (7), and Gd-PEG-Bi NPs (40). These control agents are considered ideal candidates for disease diagnosis and even treatment. Many studies have reported that DOTA and its derivatives (such as Gd-DOTA, Gd-5-HT-DOTAGA, and Gd-DO3A) have been fabricated for biological applications (46, 47, 49), which offer a unique opportunity to explore the role of these agents based on DOTA or its derivatives in the development and progression of some diseases.

Compared with the above studies, we developed Bi-DOTA to be utilized for X-ray imaging of urography and GI tract imaging. Cytotoxicity assessment revealed there was little influence on LO2, MCF-10A, HIBEC, and HUVEC cell proliferation even treated with 600 mg/L of Bi-DOTA. Meanwhile, the rapid renal clearance ability also guaranteed the low cytotoxicity of Bi-DOTA. *In vivo* toxicity evaluation (H&E staining of main organs after administration of Bi-DOTA) further proved the outstanding biosafety of Bi-DOTA, indicating its potential *in vivo* application. In our study, *in vitro* CT imaging showed that CT values of Bi-DOTA were higher than iohexol at equivalent concentrations regardless of the energy due to better X-ray attenuation ability. Furthermore, *in vivo* CT imaging also indicated the low dosage of Bi-DOTA (0.1 mol/L) enabled the acquirement of more high-quantity CT images compared with iohexol, demonstrating the superior imaging capability of Bi-DOTA. For *in vivo* GI tract, Bi-DOTA-delineated GI lumen showed remarkably enhanced signals at any energy (40–180 keV). In a word, our study clearly demonstrated the tremendous potential of Bi-DOTA as an excellent contrast agent for CT imaging owing to its promising advantages.

Of course, our study had several limitations. Considering the primary biotoxicity investigation and simple mice models used in our study, we will devote great efforts to further evaluate the long-term toxicity and CT imaging capability of Bi-DOTA in mammals and primates systematically, and promote the potential clinical implementation of Bi-DOTA in the future. Moreover, the imaging capabilities of Bi-DOTA are limited to GI tract and kidney, whose performance may be inferior to that of iohexol on other tissues. Therefore, we will develop new Bi-based small molecular contrast agents to image other tissues such as the brain, liver, and pancreas in the future. In addition, other new agents based on Bi will be fabricated and tried for CT imaging and even magnetic resonance imaging (MRI). Furthermore, future control agent could combine multiple functions into a single molecule for CT/MRI imaging and even therapy.

## Conclusion

In this study, we reported a novel small molecular Bi-DOTA complex with a facile one-step synthesis approach. The prepared control agent showed outstanding water solubility, lower cytotoxicity, and superior X-ray attenuation for *in vivo* CT imaging. These results show that Bi-DOTA will have a great potential to be a control agent for spectral CT imaging in the near future.

## Data Availability Statement

The original contributions presented in the study are included in the article/**Supplementary Material**. Further inquiries can be directed to the corresponding authors.

## Ethics Statement

The animal study was reviewed and approved by the Ethical Committee of the Affiliated Hospital of Southwest Medical University.

## Author Contributions

GD: methodology and acquisition of data. YZ: methodology and acquisition of data. XMW, XYW, JJ, and FJ: acquisition of data, investigation, and validation. LY and CY: conceptualization, analysis of data, study draft preparation, writing, reviewing, and editing. All authors listed have made a substantial, direct, and intellectual contribution to the work and approved it for publication.

## Funding

This work was supported by the NSFC (81903460), the Open Program of Nuclear Medicine and Molecular Imaging Key Laboratory of Sichuan Province (HYX19004), the Technology Strategic Cooperation Project between Luzhou Municipal People’s Government and Southwest Medical University (2020LZXNYDJ42), the Project of Southwest Medical University (2018ZRZD008, 2020ZRQNA041), and the Project of the Affiliated Hospital of Southwest Medical University (18107).

## Conflict of Interest

The authors declare that the research was conducted in the absence of any commercial or financial relationships that could be construed as a potential conflict of interest.

## Publisher’s Note

All claims expressed in this article are solely those of the authors and do not necessarily represent those of their affiliated organizations, or those of the publisher, the editors and the reviewers. Any product that may be evaluated in this article, or claim that may be made by its manufacturer, is not guaranteed or endorsed by the publisher.
