# Supplementary material for: Small-Molecule Bi-DOTA Complex for High-Performance CT and Spectral CT Bioimaging
Source: Front Oncol. 2022 Feb 18;12:813955. doi: 10.3389/fonc.2022.813955 (PMC8894608; doi:10.3389/fonc.2022.813955)
Supplement: Supplementary file 1 [file DataSheet_1.docx]

Supplementary Material

**Title: Small-Molecule Bi-DOTA Complex for high-performance spectral CT Bioimaging**


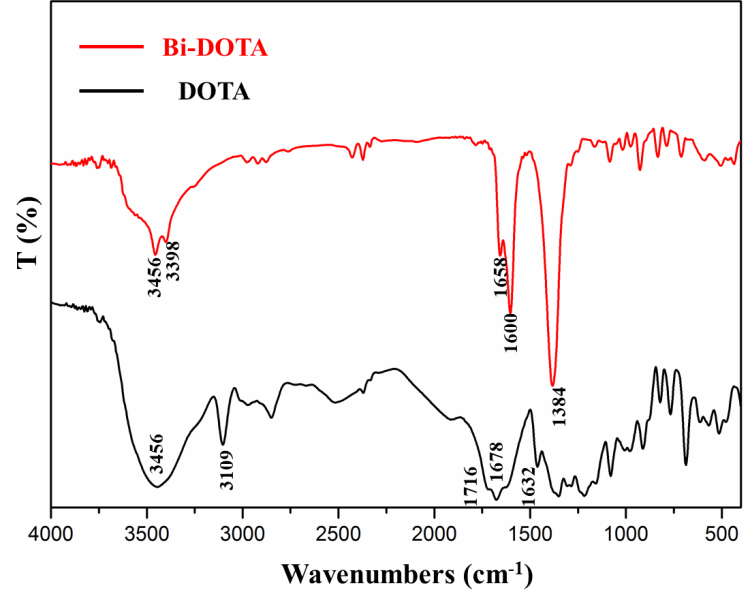


**Figure S1** FTIR spectra of DOTA and Bi-DOTA.


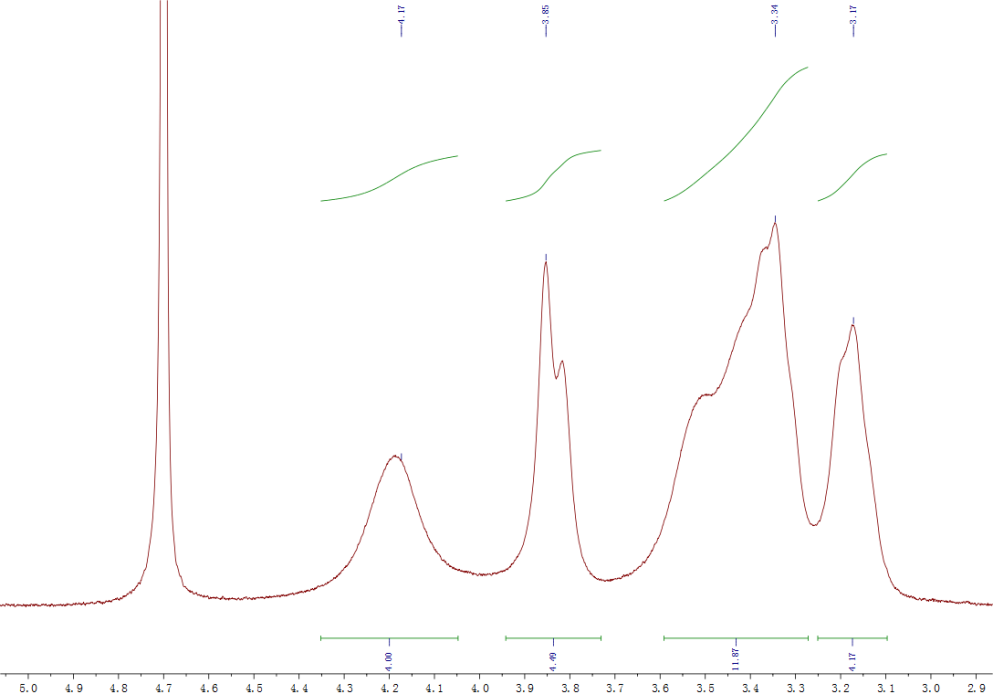


**Figure S2** ^1^H NMR (400 MHz) spectrum of Bi-DOTA (without the treatment of NaOH) in D_2_O. ^1^H NMR (400 MHz, D_2_O) *δ* 4.17 (s, 4H), 3.85 (s, 4H), 3.34 (s, 12H), 3.17 (s, 4H).


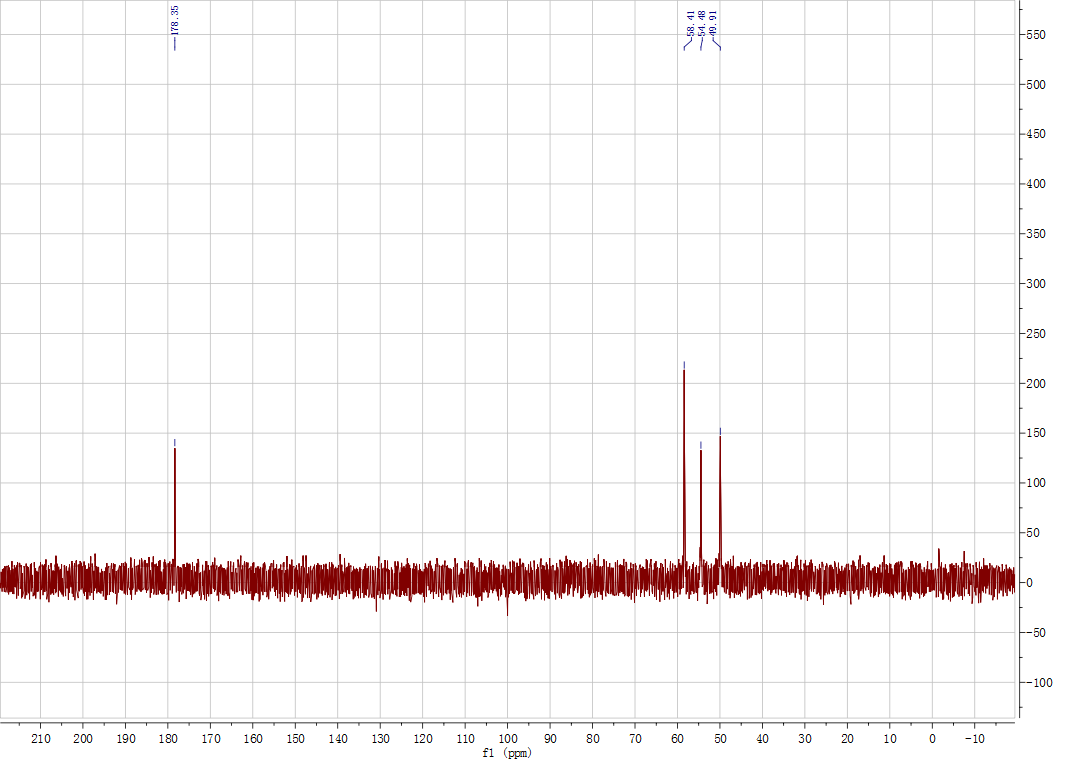


**Figure S3** ^13^C NMR (101 MHz) spectrum of Bi-DOTA in D_2_O. ^13^C NMR (101 MHz, D_2_O) *δ* 178.35, 58.41, 54.48, 49.91.


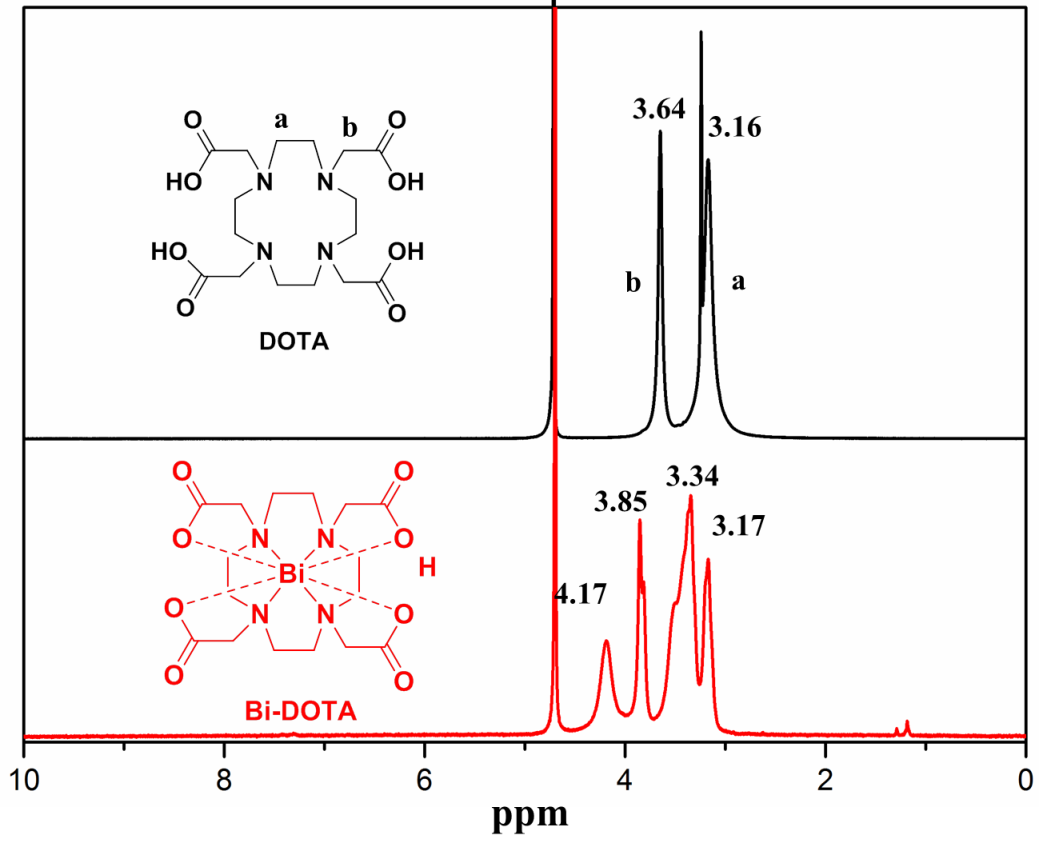


**Figure S4** ^1^H NMR (400 MHz, D_2_O) spectra of DOTA and Bi-DOTA. These results show significant changes when the -COOH of DOTA is coordinated with Bi (III) compared to DOTA. The peaks around 4.17, 3.85, 3.34 and 3.17 ppm for Bi-DOTA can be attributed to the coordination of the lanthanide center, while the peaks for DOTA are at 3.63 and 3.16 ppm.


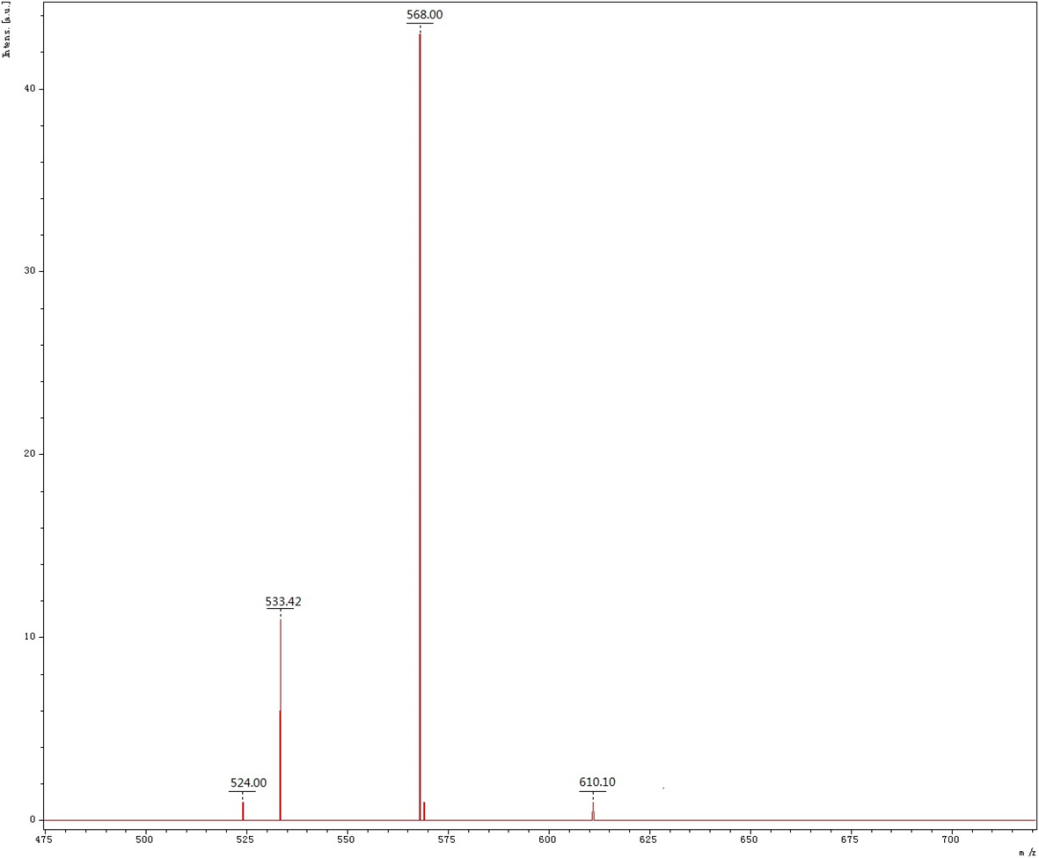


**Figure S5** MALDI-TOF-MS of Bi-DOTA. MALDI-TOF-MS Calcd for: C_16_H_25_BiN_4_O_8_^+^ ([M+H]^+^): 610.15. Found: 610.10.


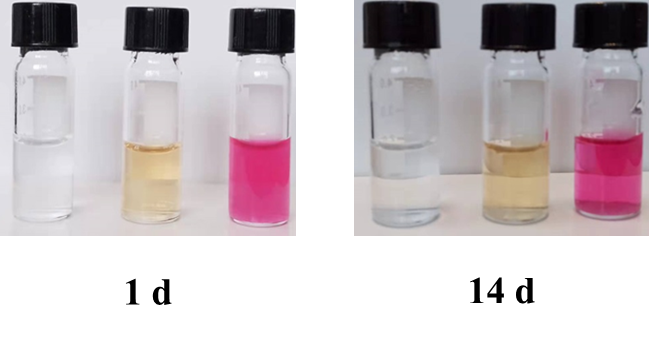


**Figure S6** The stability of Bi-DOTA in different media (100 mg/mL, from left to right: PBS, FBS and DMEM) at 37°C for 1 and 14 days.


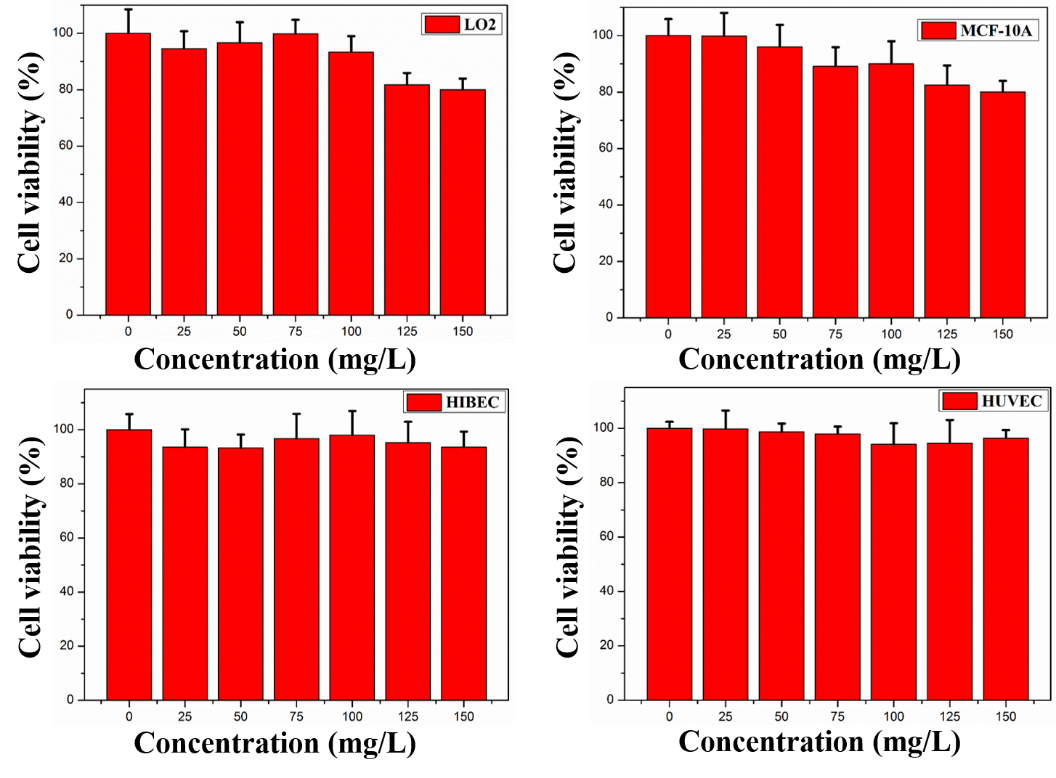


**Figure S7** In vitro relative cell viabilities of LO2, MCF-10A, HIBEC and HUVEC cells after incubation with various concentrations of Bi-DOTA.


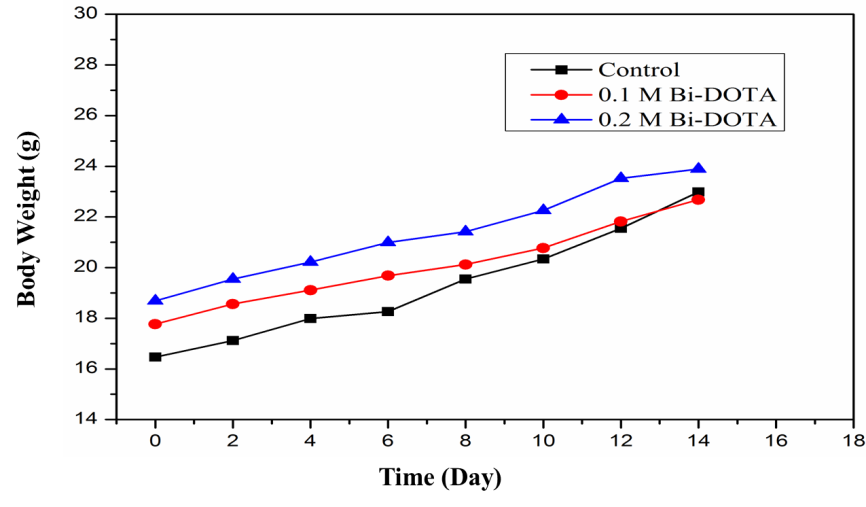


**Figure S8** The changes in body weight in different treatment groups measured every 2 days.


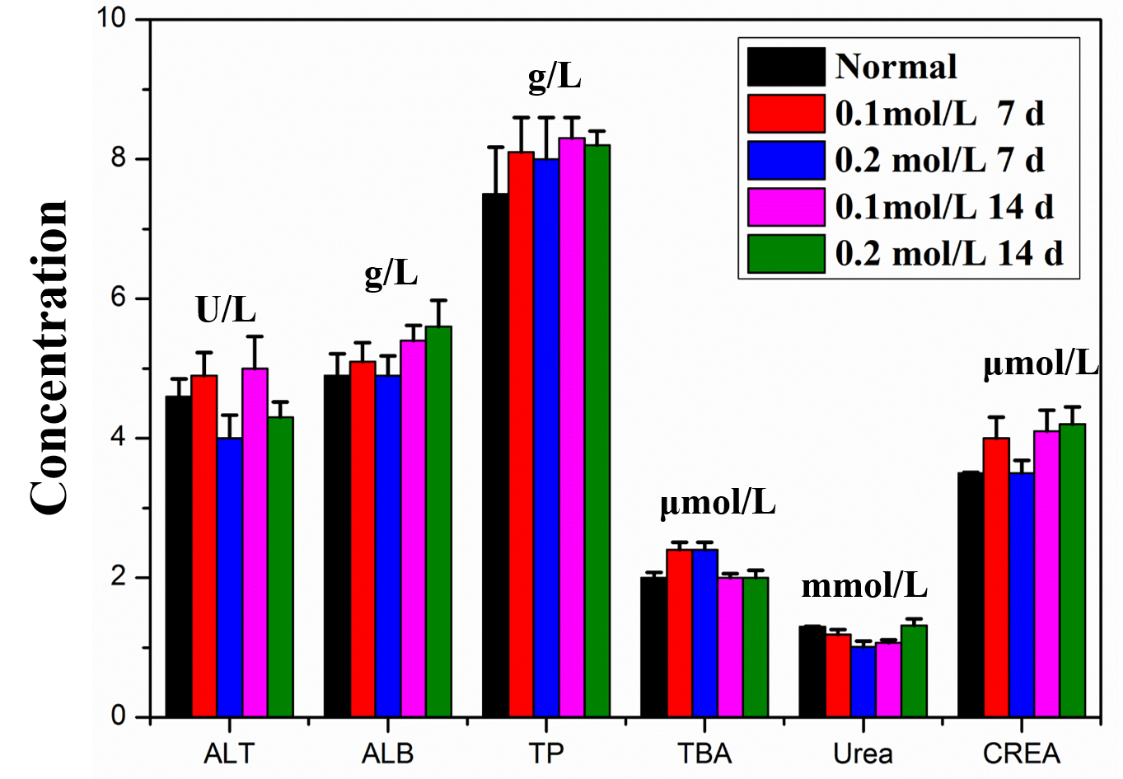


**Figure S9** Biochemical markers of mice at various time points (7 and 14 days) after intravenously administration of 200 μL Bi-DOTA (0.1 M and 0.2 M).


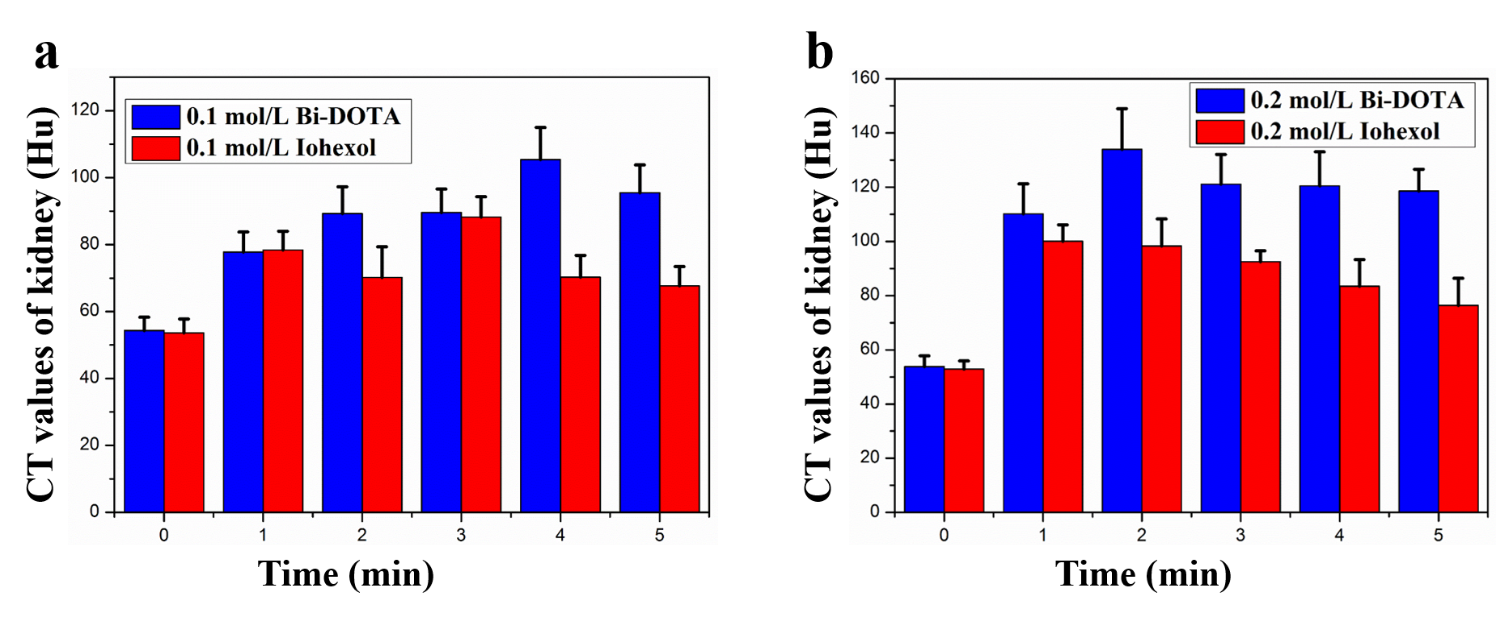


**Figure S10** CT values of kidney after administration of Bi-DOTA and Iohexol at different time points. (a) CT values of kidney after intravenous administration of 200 μL of 0.1 M Bi-DOTA and Iohexol at different time points. (b) CT values of kidney after intravenous administration of 200 μL of 0.2 M Bi-DOTA and Iohexol at different time points.
